# A large-scale genomic approach affords unprecedented resolution for the molecular epidemiology and evolutionary history of contagious caprine pleuropneumonia

**DOI:** 10.1186/s13567-015-0208-x

**Published:** 2015-07-06

**Authors:** Virginie Dupuy, Axel Verdier, François Thiaucourt, Lucía Manso-Silván

**Affiliations:** CIRAD, UMR CMAEE, F-34398 Montpellier, France; INRA, UMR1309 CMAEE, F-34398 Montpellier, France

## Abstract

Contagious caprine pleuropneumonia (CCPP), caused by *Mycoplasma capricolum* subsp. *capripneumoniae* (Mccp), is a devastating disease of domestic goats and of some wild ungulate species. The disease is currently spreading in Africa and Asia and poses a serious threat to disease-free areas. A comprehensive view of the evolutionary history and dynamics of Mccp is essential to understand the epidemiology of CCPP. Yet, analysing the diversity of genetically monomorphic pathogens, such as Mccp, is complicated due to their low variability. In this study, the molecular epidemiology and evolution of CCPP was investigated using a large-scale genomic approach based on next-generation sequencing technologies, applied to a sample of strains representing the global distribution of this disease. A highly discriminatory multigene typing system was developed, allowing the differentiation of 24 haplotypes among 25 Mccp strains distributed in six genotyping groups, which showed some correlation with geographic origin. A Bayesian approach was used to infer the first robust phylogeny of the species and to date the principal events of its evolutionary history. The emergence of Mccp was estimated only at about 270 years ago, which explains the low genetic diversity of this species despite its high mutation rate, evaluated at 1.3 × 10^−6^ substitutions per site per year. Finally, plausible scenarios were proposed to elucidate the evolution and dynamics of CCPP in Asia and Africa, though limited by the paucity of Mccp strains, particularly in Asia. This study shows how combining large-scale genomic data with spatial and temporal data makes it possible to obtain a comprehensive view of the epidemiology of CCPP, a precondition for the development of improved disease surveillance and control measures.

**Electronic supplementary material:**

The online version of this article (doi:10.1186/s13567-015-0208-x) contains supplementary material, which is available to authorized users.

## Introduction

Contagious caprine pleuropneumonia (CCPP) is a severe respiratory disease affecting goats and some wild ruminant species. The disease, listed by the World Organisation for Animal Health (OIE) [1] has a great economic impact on livestock production in fragile rural economies and poses a serious threat to disease-free areas. CCPP is caused by *Mycoplasma capricolum* subsp. *capripneumoniae* (Mccp), a member of the *Mycoplasma mycoides* cluster [2]. This cluster comprises five mycoplasmas which are pathogenic for ruminants, including *Mycoplasma capricolum* subsp. *capricolum* (Mcc), the closest relative of Mccp, and *Mycoplasma mycoides* subsp. *mycoides* (Mmm), the agent of contagious bovine pleuropneumonia (CBPP). Since it was first isolated in 1976 [3], Mccp has only been isolated in 17 countries, mainly because of its fastidiousness in culture. However, clinical descriptions have been published in nearly 40 countries in Africa and Asia, suggesting a much wider distribution [4]. The disease is present in the Arabian Peninsula, North, Central and East Africa and Asia, but its boundaries are still uncertain, particularly in western and southern Africa and in Asia. In the last decade, an increasing number of outbreaks have been reported in both domestic and wild ruminants [5]. New detections were often the result of improved diagnosis, confirming the presence of the disease in suspected regions [6–8], but in certain cases, they indicated that the disease had spread to new territories [9,10].

A comprehensive view of the evolutionary history and the dynamics of Mccp strains is essential to understand the epidemiology of CCPP. Molecular epidemiology investigations require robust genotyping tools with sufficient resolution, while evolutionary analyses are also constrained by the need for a reliable molecular clock to infer evolutionary timescales. Various molecular methods have been developed for the analysis of Mccp strains. The first study on the molecular evolution of Mccp was based on the 16S rRNA gene [11], which provided a basis for the phylogeny and systematics of bacteria since the evolutionary studies of Woese [12]. Mccp strains showed a surprisingly high degree of polymorphism between their two 16S rRNA gene copies [13], which allowed the use of this housekeeping gene to combine epidemiological and evolutionary analyses. Still, the study was limited by the low discriminatory power of this molecular marker. Despite the heterogeneity observed in the sequence of its two rRNA operons, the Mccp genome has been shown to be rather monomorphic. Thus, a phylogenic study of the *M. mycoides* cluster based on five partial housekeeping gene sequences showed a very low distance between Mccp strains [14]. The low diversity of such a monomorphic bacterium precluded the use of standard multilocus sequence typing (MLST), which relies on housekeeping genes [15]. Approaches based on alternative sequences, independently of their coding capacity, were thus preferred. Analysis of the H2 locus allowed the discrimination of four groups showing a good correlation with geographic origin [16]. This system was improved by the addition of seven loci, increasing the discriminatory power and leading to the description of five groups [4]. Although this multilocus sequence analysis (MLSA) scheme targets polymorphic loci by focusing on non-housekeeping genes (e.g., intergene regions, pseudogenes) it interrogates less than 1% of the genome and therefore has limited discriminatory power for epidemiological investigations. Moreover, it is not reliable for phylogenetic analyses because of differences in the molecular evolutionary clock among target sequences.

These limitations can now be overcome by the recent development of high-throughput methods [17]. Even though single nucleotide polymorphism (SNP) frequencies are low in monomorphic pathogens, their numbers can be dramatically increased by enlarging the scale of the analysis. Using high-throughput data can enhance genetic investigations by providing a way to disclose genome-wide variations. Combining large-scale genomic data with spatial and temporal data already enabled a comprehensive view of the molecular epidemiology and evolution of bacterial pathogens like *Salmonella typhi* [18], *Yersinia pestis* [19], Mmm [20] and *Mycobacterium tuberculosis* [21]. Following the increasing use and affordability of DNA sequencing with next-generation sequencing (NGS) technologies, complete annotated genomes of several Mccp strains have become available [22–24], thereby making large-scale genomic investigations possible.

In this study, we investigated the molecular epidemiology and evolution of CCPP using a large-scale genomic approach based on NGS data, on a sample of strains representing the global distribution of this disease. Our main objective was to develop a discriminatory genotyping method to investigate the genetic diversity and population structure of Mccp. A robust phylogeny was also inferred from a large phylogenomic data set and divergence time of Mccp strains was estimated to reconstruct the evolutionary history of CCPP.

## Materials and methods

### Sampling

The 25 strains analysed in this study are summarised in Table 1. This collection includes 15 strains isolated in Central (4)/East (11) Africa, four strains from the Arabian Peninsula, four strains from the Mediterranean Basin and two strains from Central/East Asia in attempts to encompass the known global diversity of this pathogen. They are all epidemiologically unrelated isolates and most of them were previously analysed by traditional genotyping systems [4,11,16].

**Table 1:** List of *Mycoplasma capricolum* subsp. *capripneumoniae* strains analysed and corresponding MLSA types

Strain	Year	Country	Location	World Region	Supplier	Accession number	MLSA type
97095-Tigray	1995	Ethiopia	Tigray	East Africa	NVI-E		1-010
99108-P1	1999	Ethiopia^a^	Tigray	East Africa	-	GenbaNK:JMJI00000000	1-010
04012	2004	Qatar	Doha, Al Wabra	Arabian Peninsula	AWWP		1-010
M79/93	1993	Uganda	East	East Africa	NVI-S		1-020
ILRI181	2012	Kenya	NK	East Africa	-	GenbaNK:LN515399	1-030
8789	1987	Chad	Karal, Dandi	Central Africa	LRVZF		2-010
94156	1994	Chad	N’Djamena	Central Africa	LRVZF		2-010
05021	2005	Sudan	Darfur, Nyala	Central Africa	VRA		2-010
95043	1995	Niger	Goure	Central Africa	LABOCEL		2-020
44 F04	2004	Turkey	Thrace	Mediterranean Basin	VLA		3-020
12002	2012	Tajikistan	Farkhor	Central Asia	CIRAD		3-020
C550/1	1991	UAE	Dubai	Arabian Peninsula	CVRL		3-030
M1601	2007	China	Gansu	East Asia	-	GenbaNK:CM001150	3-010
Gabes	1980	Tunisia	Gabes	Mediterranean Basin	CIRAD		4-010
9081-487P	1990	Oman	NK	Arabian Peninsula	MAF-O		4-010
07033	2007	Turkey	Elazig	Mediterranean Basin	FU		4-010
7/2	1986	Turkey^a^	NK	Mediterranean Basin	MRI		4-020
97097-Errer	1997	Ethiopia	Errer	East Africa	NVI-E		5-010
Yatta B	1997	Kenya	Yatta	East Africa	NVI-S		5-020
AMRC-C758	1981	Sudan	NK	East Africa	AU		5-020
F38	1976	Kenya	NK	East Africa	Type strain		5-030
94029-C5	1994	Oman	NK	Arabian Peninsula	AVS		5-040
91039-C3	1991	Ethiopia	NK	East Africa	NVI-E		5-050
9231-Abomsa	1982	Ethiopia	Godjam	East Africa	-	GenbaNK:LM995445	5-060
92138-CLP1	1992	Ethiopia	NK	East Africa	NVI-E		5-060

^a^Strain 99108-P1 was isolated in Eritrea, 7/2 was isolated in Oman [56] but the animals came from the location indicated above

*Abbreviations:* *AU* Aarhus University, Denmark, *AVS* Agriculture and Veterinary Services, Oman, *AWWP* Al Wabra Wildlife Preservation, Qatar, CIRAD, France, *CVRL* Central Veterinary Research Laboratory, United Arab Emirates, *FU* Firat University, Turkey, *LABOCEL* Laboratoire Central de l’Elevage de Niamey, Niger, *LRVZF* Laboratoire de Recherches Vétérinaires et Zootechniques de Farcha, Chad, *MAF-O* Ministry of Agriculture and Fisheries, Oman, *MRI* Moredun Research Institute, UK, *NVI-E* National Veterinary Institute, Ethiopia, *NVI-S* National Veterinary Institute, Sweden, *VRA* Veterinary Research Administration, Sudan, *NK* not known

### Sample preparation and sequencing

Twenty-one strains for which the genome sequence was not available were cultured in modified Hayflick’s medium [14] at 37 °C, 5% CO_2_. Culture purity was ensured by phenotypic control on solid medium and specific Mccp QPCR amplification [25]. DNA was extracted using a standard phenol/chloroform method [26]. DNA purity, quality and quantity were checked using NanoDrop™ ND-1000 Spectrophotometer (Thermo Fisher Scientific, MA, USA), gel electrophoresis and Qubit® 2.0 fluorometer (Invitrogen, USA), respectively. Then, 21 tagged standard genomic libraries were constructed and pooled to be sequenced in 100 bp single reads on an Illumina HiSeq 2000 (GATC, Constanz, Germany).

### Gene selection

The choice of Mccp genes was based on the choice previously made for the analysis of the evolutionary history of Mmm [20], consisting in 62 rigorously selected genes. Pseudogenes and duplicated genes had been excluded from this set, as well as genes coding for membrane proteins or restriction enzymes, and those known to be involved in horizontal transfer. The annotated, circularised genome sequence of strain 9231-Abomsa [23] (Table 1) was used as reference. Four genes did not exist in the Mccp genome (*guaC, gntR, suk, bgl*), while *dnaC* was duplicated and was therefore excluded. As a result, a subset of 57 genes, comprising 47 coding sequences and 10 pseudogenes of the core genome, were used (Additional file 1). The 57 genes are evenly distributed along the chromosome of strain 9231-Abomsa (Additional file 2).

### Data set collection

The sequences of the 57 selected genes from strain 9231-Abomsa, including flanking regions (up to 350 bp), were concatenated and annotated, resulting in an “enlarged” sequence of 107 050 bp (Additional file 3). This sequence was used as reference to automatically retrieve the entire gene set from the whole genome sequence data of 21 Mccp strains by mapping raw data using Seqman NGen (2.0) software (DNASTAR, Madison WI, USA). First, this procedure allowed the correct mapping of reads on the entire sequence of each corresponding gene, thanks to the presence of flanking regions. Second, it allowed the visual verification of sequencing depth and the identification of any incongruities on all coding sequences in the Seqman genome browser. On average, a read depth of 500X was obtained. SNPs and indels were called when more than 85% of the reads supported the change.

An in-house software was developed at CIRAD Montpellier to extract and concatenate the gene sequences corresponding to each strain from the enlarged reference sequence. Sequence searches are based on the Needleman-Wunsch algorithm, using tags to frame sequences of interest. These tags are short sequences homologous to the extremities of each target gene. The algorithm described here is implemented in C++ as a stand-alone program, “SelectRegion”, and the source is freely available from the authors on request. The process can also be automated using graphical-interfaces within the web-based Galaxy [27].

Otherwise, sequence data were retrieved from the published genome sequences of strains M1601, 99108 and ILRI181 (Table 1).

### Genotyping analyses

For diversity analyses, the 57 genes (comprising both coding sequences and pseudogenes) corresponding to each of the 25 strains analysed were selected and concatenated as described above. Sequences were aligned using Clustal W with default parameters (Additional file 4). Haplotypes were estimated taking into account all sites, including gaps, removing invariant sites, with DnaSP [28]. A median-joining network was reconstructed using NETWORK V4.6 [29].

The discriminatory power of the genotyping system was calculated using Simpson’s index of diversity [30], which expresses the probability of two unrelated strains sampled from the test population being placed into different genotyping groups. A total of 95% confidence intervals (CI) were determined as previously described [31].

### Phylogeny and molecular dating analyses

From the 57 genes initially selected, a subset of 47 coding sequences was retained for evolutionary analyses. The 47 genes of 25 Mccp strains were extracted from each corresponding enlarged reference sequence using the internal software SelectRegion. The type strain of the subspecies Mcc (California Kid^T^), which is the closest relative of Mccp, was chosen as outgroup and corresponding sequence data were obtained from the published genome sequence [Genbank:CP000123].

Sequences were aligned using Clustal W with default parameters (Additional file 5). The Tamura-Nei, 93 (TN93) model was selected as the best fitting model by Modeltest V3.7 [32]. A maximum-likelihood phylogenetic tree was inferred using PhyML V3.0 [33] on the Galaxy web platform. A bootstrap resampling procedure with 1000 replicates was used to assess the reliability of key tree nodes. To infer a temporal framework from dated sequences, a Bayesian approach, implemented in the flexible Bayesian phylogenetic analysis package BEAST V1.6.2 [34], was used. This allowed the simultaneous estimation of the tree structure, the time of the most recent common ancestor (MRCA), the divergence time of nodes, and the mutation rate. The TN93 evolution model and partition codon (1 + 2, 3) were selected. Two molecular clocks (strict-clock and uncorrelated lognormal-clock) and various demographic models (constant, expansion, exponential and extended Bayesian skyline plot) were tested. Convergence was evaluated using Tracer V1.6. To choose the best fitting clock and demographic model, the Bayes Factors [35,36] were calculated from the marginal likelihoods on the Akaike’s information criterion through MCMC (AICM) using Tracer V1.6. The Maximum Clade Credibility tree was constructed using TreeAnnotator V1.6 and visualised using FigTree V1.3.

## Results

### Genetic diversity and molecular genotyping of Mccp strains

A discriminatory genotyping system based on a large-scale genomic approach was developed to characterise the diversity of Mccp by analysing 25 strains representing the known global distribution of this species (Table 1). A set of 57 genes, comprising 47 coding sequences and 10 pseudogenes (Additional file 1), was analysed, covering 77 898 base pairs (7.7% of the genome). Fifty-two of these genes were polymorphic as a result of 239 polymorphic positions consisting in 212 SNPs and 17 indels (Additional file 6). Nine events corresponded to indels of either one/two bases or one/two codons and eight events to variations in homopolymer size. The average frequency of polymorphic sites in the gene set was of one event per 577 pb for coding sequences, and one event per 210 pb for pseudogenes. The 239 polymorphic positions made it possible to define 24 haplotypes among the 25 strains analysed, resulting in a Simpson’s diversity index of 0.997 (0.988-1.000). Polymorphic positions consisted in 115 informative sites and 124 sites specific to single haplotypes.

**Figure 1:**
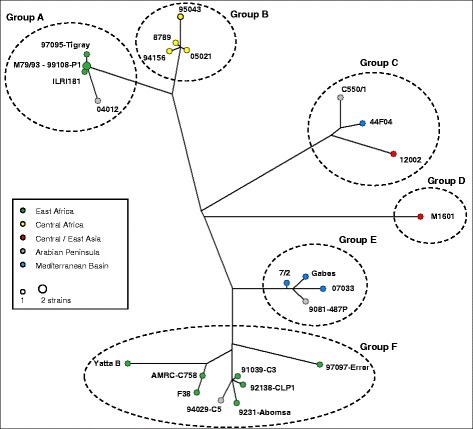
**Haplotype network of** ***Mycoplasma capricolum*** **subsp.** ***capripneumoniae.*** The median-joining network was reconstructed using NETWORK based on the alignment of 57 concatenated genes from 25 Mccp strains. The tree shows 24 haplotypes (nodes) resulting from 239 polymorphic positions including gaps. Different colours indicate the geographic origin of haplotypes.

A single network connecting all strains was drawn using NETWORK (Figure 1). The 24 haplotypes were structured in six genotyping groups, named group A to F. Group A was quite homogeneous and included four extremely similar strains from East Africa and a more distant strain from the Arabian Peninsula. Within this group, M79/93 and 99108-P1 were the only strains that could not be distinguished. Four strains from Central Africa, also showing little diversity, constituted group B. Group C included three rather variable strains from the Mediterranean Basin, the Arabian Peninsula and Central Asia. Chinese strain M1601, positioned at the extremity of a long isolated branch, was designated group D, and was the only representative of this group. Group E showed little variability and comprised three strains originating from the Mediterranean Basin and a strain from the Arabian Peninsula. Finally, Group F, which was the most populated and variable group, included seven strains from East Africa and one strain from the Arabian Peninsula.

Three groups (A, B and F) presented at least four isolates originating from the same geographic region, whereas too few strains from groups D and E were available to confirm a correlation between genotyping group and geographic origin. In group C no clear correlation could be found with geographic origin, also arguably due to insufficient sampling. In the Arabian Peninsula many different groups (all except B and D) were found and in Turkey two groups (C and E) were present. Also, two distinct groups (A and F) were found in East Africa.

### Evolutionary history of Mccp

**Figure 2:**
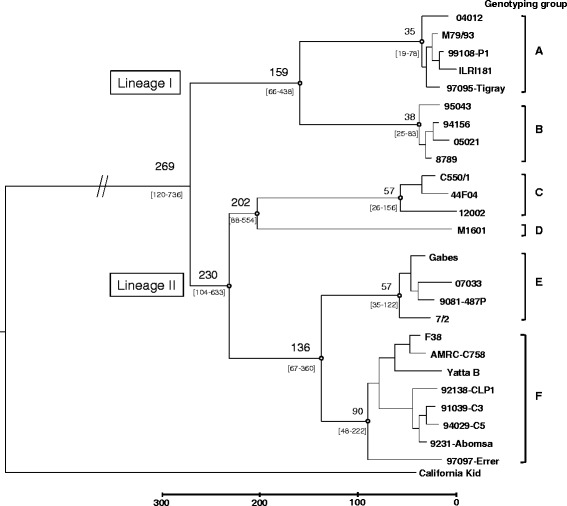
**Bayesian inference of** ***Mycoplasma capricolum*** **subsp.** ***capripneumoniae*** **evolutionary history.** The Maximum Clade Credibility tree resulted from BEAST analysis of the alignment of concatenated sequences of 47 coding sequences from 25 Mccp strains. Bayesian posterior probabilities higher than 0.9 are represented by a white circle. Age estimates are displayed for relevant nodes with the 95% highest probability density intervals in brackets. The scale is given in years before present. The branch corresponding to the outgroup (California Kid) was shortened, as indicated by parallel bars.

A robust phylogeny of Mccp was reconstructed based on high-throughput genomic data of 25 selected Mccp strains (Table 1) and an Mcc outgroup. Among the 57 genes previously analysed for genotyping, a set of 47 coding sequences was retained, while pseudogenes were excluded (Additional file 1) to minimise molecular clock variation and homoplasy. After alignment of the sequences, robust trees were inferred based on 134 SNPs using both maximum likelihood (Additional file 7) and Bayesian (Figure 2) approaches. Tree topologies were identical for supported branches. The six genotyping groups described above (group A to group F) were retrieved as clusters supported by high node support values. They formed two major lineages. Lineage I included group A, present in East Africa, and group B, present in Central Africa. Lineage II comprised groups C, D, E and F and was divided into two sub-lineages. The first sub-lineage comprised groups C and D, representing Asian strains, while group E (representing strains from the Mediterranean Basin) and group F (comprising mainly strains from East Africa) were clustered in a second, heterogeneous sub-lineage. Divergence time for Mccp strains was estimated from the dated sequences using the flexible Bayesian phylogenetic analysis package BEAST (Figure 2). Bayes Factor tests indicated that the strict-clock model fitted the data better than the relaxed-clock model. In contrast, there was no substantial difference between population models. To exempt analyses from dependence on a pre-specified demographic model, the extended Bayesian skyline plot model was applied. The mean substitution rate for Mccp, estimated on 47 coding sequences, was 1.3 × 10^−6^ (substitutions per site per year). The MRCA of Mccp strains emerged 269 years ago (95% highest posterior densities (HPDs) intervals between 120 years and 736 years). Lineage II diverged around 230 years ago (95% HPDs: 104–633) and dissociated into two sub-lineages dated 202 (95% HPDs: 88–554) and 136 years ago (95% HPDs: 67–360) respectively, while lineage I diverged around 159 years ago (95% HPDs: 66–438). All genotyping groups except D were formed recently at almost the same period (between 19 and 222 years ago), with the most probable age being between 35 years (for group A) and 90 years (for group F). Indeed, group D is represented by a single strain (the only Chinese strain available so far) and, as long as the diversity of this group remains unresolved, it will not be possible to date its emergence.

## Discussion

### Increasing the scale of analysis to tackle the genetic population structure and evolution of the monomorphic pathogen Mccp

Analysing the diversity in genetically monomorphic pathogens such as Mccp is a challenge because of their low variability. Classical molecular approaches based on sequencing a few gene fragments provide so little appreciable diversity that, to date, few questions regarding the epidemiology and evolution of CCPP have been answered. Technological advances in DNA sequencing now allow the rapid sequencing of complete bacterial genomes and offer the possibility to multiplex many different isolates, thereby dramatically increasing the discovery of polymorphic positions to analyse species diversity [37]. In this study, using data from NGS technologies made it possible to develop new approaches based on the analysis of a large number of genes.

The choice of genes for Mccp was based on a recent study of the evolutionary history of Mmm [20]. This pathogen, responsible for CBPP, is also a very monomorphic species for which classical methods like MLST do not afford sufficient discriminatory power. In this previous study, the rigorous selection of 62 genes from the core genome allowed the differentiation of 19 genotypes out of 20 strains analysed and made it possible to elucidate the evolutionary history of CBPP. Therefore, the same approach was chosen to investigate the molecular epidemiology and evolution of CCPP. In the present study, the initial set of 62 genes was reduced to 57 genes after exclusion of missing and duplicated genes in Mccp. The selection finally comprised 47 coding sequences and 10 pseudogenes of the core genome. However, it should be noted that the complete set of 57 genes was only used to conduct genotyping analysis. Keeping the pseudogenes in this gene set gave the genotyping system increased discriminatory power. Indeed, because of their neutral evolution, pseudogenes benefit from a much higher rate of nucleotide substitution than coding sequences [38]. As a result, in our study, the inclusion of pseudogenes made it possible to distinguish strains that could not be distinguished in the phylogenetic analysis based only on 47 coding sequences. Furthermore, almost 20% of the selected Mccp genes were pseudogenes, implying that the analysis of new strains in future genotyping studies may possibly disclose additional pseudogenisation events. On the other hand, for evolutionary analysis, all pseudogenes were discarded. Precise phylogenetic inference and reliable molecular dating were thus based on 47 coding gene sequences presenting similar rates of evolution. Furthermore, detailed sequence analysis did not reveal any mutation hotspots that may have suggested inter-specific recombination. Therefore, the set of genes was considered appropriate for reliable evolutionary analysis.

This multigene approach was applied to a sample of 25 epidemiologically unrelated strains, representing the known global diversity of this species. A large number of stable molecular markers were then selected from this sample by fixing the threshold of polymorphic positions to 85%, therefore excluding potential hypervariable positions. The strategy used here to identify polymorphic positions, based on the comparison of multiple independent strains representing the global species diversity, made it possible to minimise the phylogenetic discovery bias [17].

In the near future, “Whole genome sequencing” (WGS) is expected to become customary for molecular genotyping, since it provides access to information on the entire genome [39]. However, to enable the use of WGS in routine diagnostic laboratories, the main challenge remains the provision of accessible bioinformatics tools to enable the extraction of relevant information out of the enormous amount of data generated, for a fast and reliable analysis [40,41]. The use of WGS data from diverse strains would also facilitate new approaches to elucidate the evolution of monomorphic species. However, for evolutionary analyses, the problem is further complicated by the need for reliable molecular clock rates [17].

### A new discriminatory system for CCPP epidemiological investigations

The genotyping system developed here improves robustness and resolution for CCPP epidemiological analysis compared to classical typing methods. The MLST method could not be applied due to insufficient discriminatory power. As an illustration, an MLST scheme based on seven partial housekeeping gene sequences used for the study of the evolution of the *M. mycoides* cluster revealed only six polymorphic positions between 14 Mccp strains [42]. Seven of these strains were included in our study in which they were distinguished by 135 polymorphisms. As an alternative to MLST, an MLSA system had been developed [4], which remained limited in terms of variability. Our new system, which includes most of the strains previously analysed by MLSA (Table 1), is based on a higher number of polymorphic positions (239 polymorphisms identified in 77 912 bp, versus 53 polymorphisms found in 6747 bp for the MLSA system), resulting in increased discriminatory power (Simpson’s index of diversity of 0.997 (0.988-1.000) versus 0.964 (0.937-0.991) for the MLSA system).

The six genotyping groups identified in this study (groups A to F; Figure 1) are basically in accordance with MLSA clustering (groups 1 to 5; Table 1) except that diversity analyses now provide evidence for an additional group represented by a single Chinese strain. This strain (M1601) was previously included in MLSA group 3, together with two other strains from the Asian continent, although it branched separately in the MLSA tree, rendering this group polyphyletic. This anomaly was previously accepted because strain M1601 was the only one in the group that did not present a large deletion of around 1000 bp, which may have biased distance analysis. However, the present analysis, which is more robust and discriminatory, called for a re-classification of this genotype in a new group (D), the distribution and variability of which remain to be characterised. Most importantly, the intra-group resolution was dramatically increased in the present system, allowing all but two of the strains analysed to be differentiated, while MLSA only afforded further distribution of strains into sub-groups, resulting in a biased presentation of the evolutionary distance between strains (Table 1).

A highly discriminatory genotyping tool is now available for the molecular epidemiology of CCPP, allowing precise epidemiological investigations. Recent works have reported the spread of CCPP to new territories, such as the Indian Ocean [9] or the Thrace region of Turkey [10], which constituted a serious threat to Europe. Also, Mccp has been isolated from an increasing variety of wild ungulate species [43–45]. This striking discovery has changed our view of the strict host specificity of Mccp and poses new challenges for CCPP surveillance and control. Tracing new outbreaks requires accurate knowledge of the genetic population structure and the geographic distribution of strains, while the main limitation to such a study remains the paucity of Mccp isolations due to the fastidiousness of this bacterium. However, the use of high throughput sequencing, applied directly on Mccp-rich pleural fluid samples, could circumvent this problem and ease the establishment of a representative Mccp genome database.

### The monomorphic bacterium Mccp: a recently emerged pathogen undergoing rapid evolution

A large-scale genomic approach made it possible to infer the first robust and discriminatory phylogeny of Mccp, to estimate its evolutionary rate, and to date its emergence. A previous study on the molecular evolution of Mccp, based on polymorphisms in the two 16S rRNA gene copies [11], only provided evidence for two major lineages for the species. Our study confirmed the presence of these two lineages but, most importantly, it enabled a dramatic increase in the resolution and robustness of the phylogenetic analysis, resulting in the identification of six well-supported clusters. In addition, this multigene approach made it possible to overcome the bias caused by single gene specificities, providing a more reliable inference of the phylogeny of Mccp [46].

The mutation rate of Mccp was estimated by BEAST at 1.3 × 10^−6^ substitutions per site per year, based exclusively on coding gene sequences. This estimate is in agreement with previous reports for other mycoplasma species [20,47], and exceeds the mutation rate estimated for Mmm at 5.0 × 10^−7^ based on practically the same gene set. *Mycoplasmas* are among the fastest evolving bacteria, which may be explained by the loss of DNA repair genes during reductive genome evolution [48]. These high mutation rates may facilitate the accumulation of nonsense and frame-shift mutations resulting in gene decay, as evidenced in the analysis of the complete genome of strain 9231-Abomsa, which revealed the presence of 248 pseudogenes representing 25% of the genome [23]. Increased fixation of detrimental mutations by Mccp may be associated with the severe population bottleneck which characterised the emergence of this species. Indeed, evolutionary and dating analysis made it possible to recreate a plausible scenario for the emergence of the species: i.e., the specialisation of Mcc, a goat pathogen presenting a wider tissue tropism, in a restricted ecological niche (the lung) resulted in the emergence of Mccp. This emergence is indeed a recent event, estimated by BEAST analysis at about 270 years ago (between 120 and 736), in agreement with the estimate of Fischer et al. [42], dating the origin of the species between 56 and 490 years ago. Mccp, like other young pathogens, has evolved too recently to allow the accumulation of many mutations [49,50], thus explaining its low genetic diversity despite a high mutation rate.

### Combining large-scale genomic data with spatial and temporal data provides an unprecedented resolution to elaborate plausible scenarios regarding CCPP evolution and epidemiology

BEAST analysis showed that the ancestor of all Mccp strains emerged about 270 years ago, around 1740. The first unequivocal accounts of CCPP were reported more than a century after this predicted emergence in Algeria [51] and then in South Africa, involving goats imported from Turkey [52]. It is therefore difficult to establish where this emergence took place simply by examining the literature, particularly since, at that time, CCPP was confused with diseases caused by other mycoplasmas of the *M. mycoides* cluster which frequently infect goats and induce similar clinical signs and lesions. Historical data must therefore be analysed very carefully to ensure that Mccp was indeed the etiologic agent.

**Figure 3:**
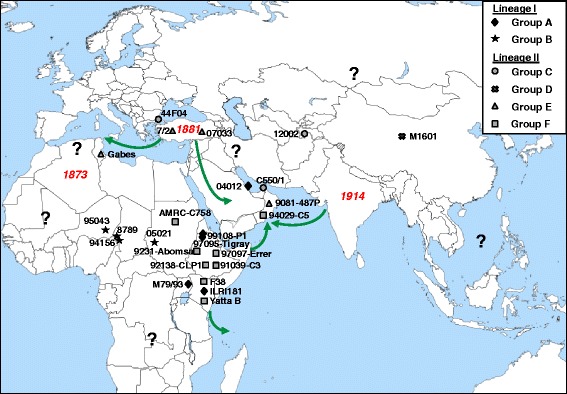
**Integration of genomic, geographic, historical and animal movement data to investigate the epidemiology of CCPP.** The original location of each Mccp strain is indicated by a symbol, according to the corresponding genotyping group. The green arrows show major CCPP diffusion routes. The dates of the first clinical descriptions of the disease are indicated in red italics. Question marks are placed in areas from where no recent data is available.

Molecular dating indicated that the MRCA of group C (including strains from Tajikistan, the Arabian Peninsula and Turkey) and group D (represented by one strain from China) (Figure 3) emerged about 200 years ago, around 1810, indicating that CCPP was present for a long time in Asia. The first historical description of CCPP on this continent may well be that made by Walker in India in 1914 [53]. Molecular dating and historical data therefore suggest that the Asian continent may be the cradle of CCPP. In the Arabian Peninsula, most of the genotyping groups were observed, representing both evolutionary lineages (Figure 3). This finding is not surprising, since this region is known to extensively import animals from various origins, particularly for Muslim feasts [16]. On the other hand, Turkey has been known to export animals to many different areas for a long time [52]. In the present study, two different groups were identified in this country (Figure 3). While the occurrence of group E in North Africa and Turkey is in agreement with animal movements reflecting well known Mediterranean trading routes established during the Ottoman Empire, the identification of group C in Turkey reveals the great complexity of animal movements in this region. The scarcity of strains from the West, but also from Central and East Asia hampers the precise determination of the emergence, diversity and distribution of Mccp in Asia.

In East Africa, two distinct groups (A and F) were found, each belonging to a different lineage (Figure 3), with MRCAs estimated around 35 and 90 years ago, respectively. Intra-group variability was extremely low for group A strains, while Group F strains showed higher genetic diversity, in agreement with longer evolution times allowing further clonal expansion. The presence of two extremely divergent groups in East Africa suggests that Mccp emerged in this region on at least two different occasions. Strains from both groups then spread thanks to trade and other animal movements between neighbouring countries. However, the origin and dynamics of co-evolution of these two distinctive groups in East Africa remain to be elucidated.

In Central Africa, a strict correlation between genotype (group B) and geographic origin was identified. In addition, the four strains belonging to this group showed very little genetic diversity and the date of their MRCA was estimated at around 40 years ago, suggesting that Mccp must have emerged very recently in Central Africa, as stated by Lefevre et al. when the first strain was isolated in Chad in 1987 [54]. In fact, CCPP was not previously suspected by veterinarians established in this region during the colonial times, although they were familiar with the typical clinical picture of this disease, which was described in scientific journals and reference works [55]. If we consider the distribution of other contagious diseases of goats, such as the “peste des petits ruminants”, the presence of CCPP may also be suspected in West Africa. An active search involving improved epidemiological surveillance networks will be required to determine the western limits of CCPP distribution in Africa.

In conclusion, this study illustrates how using a large scale genomic approach can provide an unprecedented resolution to analyse the dynamics and evolution of Mccp. Combining high-throughput genomic data with spatial and temporal data enabled a comprehensive view of the epidemiology of CCPP, which will facilitate the development of improved disease surveillance and control measures. Further efforts are now needed to better define the genetic diversity and distribution of Mccp, particularly in North and Central Africa and in Asia, from where very few strains have been isolated to date.

## Additional files

Additional file 1:
**List of the 57 genes analysed according to the genome of Mccp strain 9231-Abomsa.** The 57 genes selected for analysis are described according to the genome of strain 9231-Abomsa. Additional file 2:
**Distribution of selected genes in the genome of Mccp strain 9231-Abomsa.** Illustration of the 57 selected genes evenly distributed along the chromosome of strain 9231-Abomsa. Additional file 3:
**Mccp strain 9231-Abomsa gene set reference sequence.** This reference sequence was used to automatically retrieve the entire gene set from NGS raw data. It consists in the 57 annotated genes from Mccp strain 9231-Abomsa separated by flanking regions (107.050 bp). Additional file 4:
**Alignment of 57 concatenated genes of 25 Mccp strains.** Input file for genotyping analysis. Additional file 5:
**Alignment of 47 concatenated coding sequences of 25 Mccp strains and an Mcc outgroup.** Input file for phylogenetic analysis. Additional file 6:
**List of polymorphic positions.** Description of the polymorphic positions found in the genotyping dataset. Additional file 7:
**Phylogeny of** ***Mycoplasma capricolum*** **subsp.** ***capripneumoniae*** **inferred with the maximum likelihood method.** The maximum likelihood tree was reconstructed using PhyML based on the alignment of the concatenated sequences of 47 coding sequences from 25 Mccp strains. Bootstrap values > 90% are shown. The branch corresponding to the outgroup (California Kid) was shortened, as indicated by parallel bars. The scale bar shows the number of substitutions per site.
