# Supplementary figures and images for: A large-scale genomic approach affords unprecedented resolution for the molecular epidemiology and evolutionary history of contagious caprine pleuropneumonia
Source: Vet Res. 2015 Jul 6;46(1):74. doi: 10.1186/s13567-015-0208-x (PMC4492101; doi:10.1186/s13567-015-0208-x)

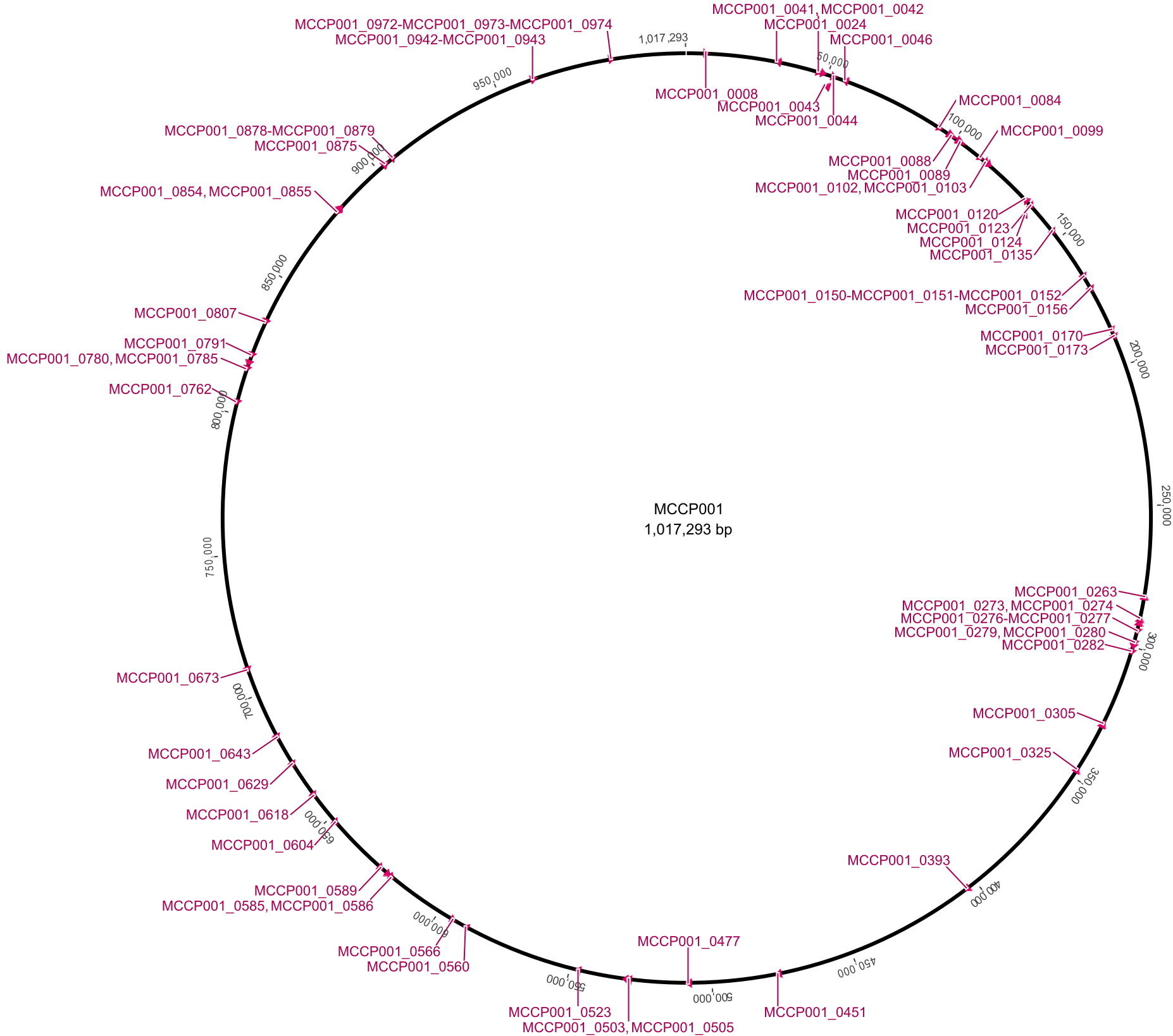

Supplement: Additional file 2: — Distribution of selected genes in the genome of Mccp strain 9231-Abomsa. Illustration of the 57 selected genes evenly distributed along the chromosome of strain 9231-Abomsa. [file 13567_2015_208_MOESM2_ESM.pdf]

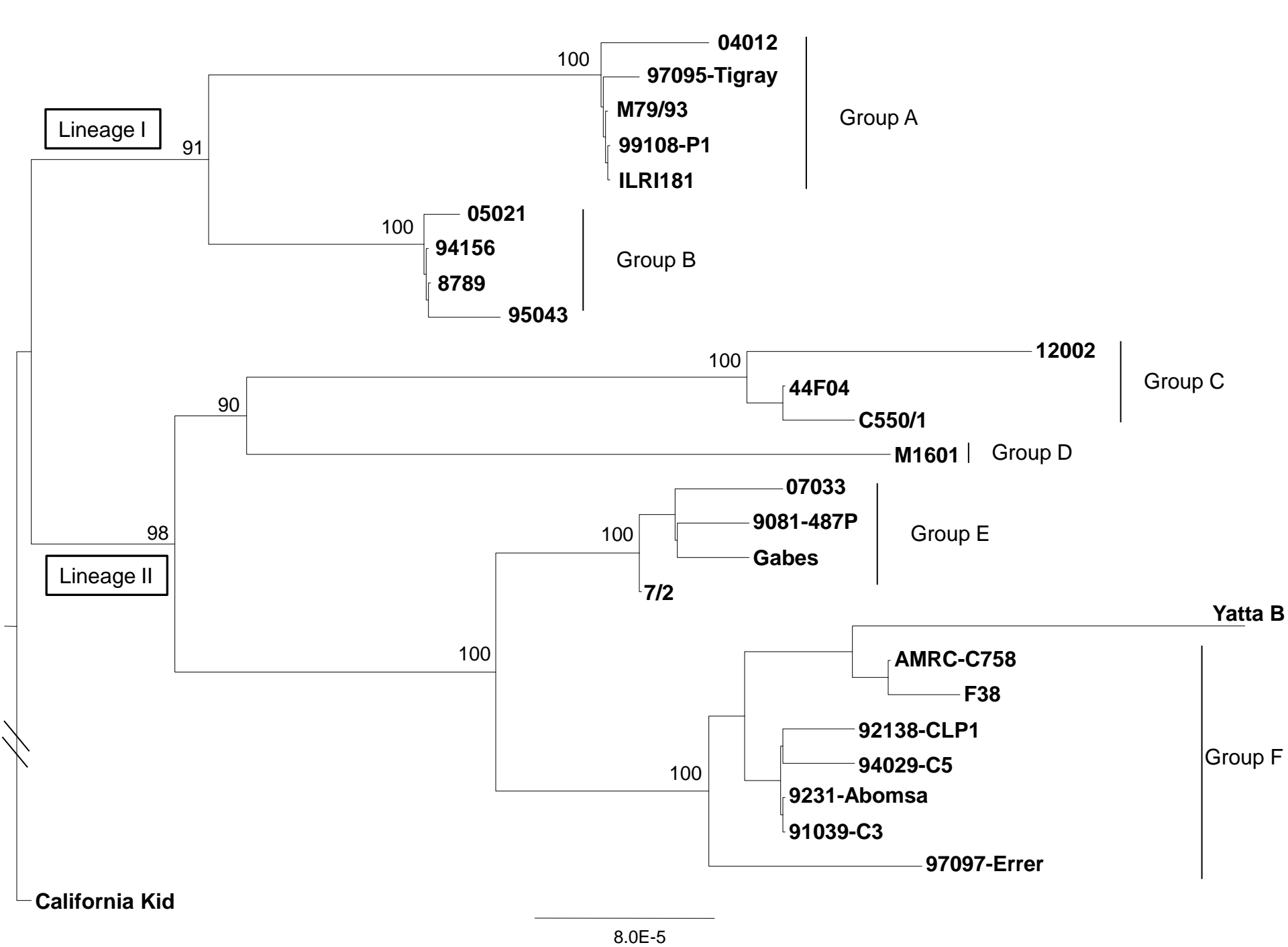

Supplement: Additional file 7: — Phylogeny of Mycoplasma capricolum subsp. capripneumoniae inferred with the maximum likelihood method. The maximum likelihood tree was reconstructed using PhyML based on the alignment of the concatenated sequences of 47 coding sequences from 25 Mccp strains. Bootstrap values > 90% are shown. The branch corresponding to the outgroup (California Kid) was shortened, as indicated by parallel bars. The scale bar shows the number of substitutions per site. [file 13567_2015_208_MOESM7_ESM.pdf]
